# Connectome-based modeling reveals a resting-state functional network that mediates the relationship between social rejection and rumination

**DOI:** 10.3389/fpsyg.2023.1264221

**Published:** 2023-10-30

**Authors:** Li Geng, Qiuyang Feng, Xueyang Wang, Yixin Gao, Lei Hao, Jiang Qiu

**Affiliations:** ^1^Key Laboratory of Cognition and Personality, Ministry of Education, Southwest University, Chongqing, China; ^2^Faculty of Psychology, Southwest University, Chongqing, China; ^3^College of Teacher Education, Southwest University, Chongqing, China

**Keywords:** rumination, social rejection, functional connectivity, prediction model, resting-state fMRI

## Abstract

**Background:**

Rumination impedes problem solving and is one of the most important factors in the onset and maintenance of multiple psychiatric disorders. The current study aims to investigate the impact of social rejection on rumination and explore the underlying neural mechanisms involved in this process.

**Methods:**

We utilized psychological questionnaire and resting-state brain imaging data from a sample of 560 individuals. The predictive model for rumination scores was constructed using resting-state functional connectivity data through connectome-based predictive modeling. Additionally, a mediation analysis was conducted to investigate the mediating role of the prediction network in the relationship between social rejection and rumination.

**Results:**

A positive correlation between social rejection and rumination was found. We obtained the prediction model of rumination and found that the strongest contributions came from the intra- and internetwork connectivity within the default mode network (DMN), dorsal attention network (DAN), frontoparietal control network (FPCN), and sensorimotor networks (SMN). Analysis of node strength revealed the significance of the supramarginal gyrus (SMG) and angular gyrus (AG) as key nodes in the prediction model. In addition, mediation analysis showed that the strength of the prediction network mediated the relationship between social rejection and rumination.

**Conclusion:**

The findings highlight the crucial role of functional connections among the DMN, DAN, FPCN, and SMN in linking social rejection and rumination, particular in brain regions implicated in social cognition and emotion, namely the SMG and AG regions. These results enhance our understanding of the consequences of social rejection and provide insights for novel intervention strategies targeting rumination.

## 1. Introduction

Rumination refers to an individual’s passive and repeated recollection, reflection, and analysis of problems in a negative emotional state and is a negative style of cognitive appraisal of painful events (Nolen-Hoeksema, 1991; Nolen-Hoeksema et al., 2008). In a recent review, Watkins and Roberts (2020) defined rumination as repetitive thoughts about the symptoms, causes, circumstances, meanings, and consequences of negative mood. Research suggests that rumination is not only associated with emotional and mental health problems such as anxiety (Olatunji et al., 2013), depression (Spasojević and Alloy, 2001), and sadness (Kirkegaard Thomsen, 2006) but may also lead to impaired memory and executive control (Davis and Nolen-Hoeksema, 2000; Watkins and Brown, 2002) as well as cognitive dissonance (through excessive attention to and memory of negative information)(Watkins and Teasdale, 2001; Whitmer and Gotlib, 2013) and impaired positive problem solving (Lyubomirsky and Nolen-Hoeksema, 1995; Watkins and Baracaia, 2002). Theories suggest that rumination is one of the key factors in the maintenance of depression and other disorders (Lyubomirsky and Nolen-Hoeksema, 1995). Rumination can exacerbate neurological disorders in several ways, including amplifying and prolonging existing negative emotions and thoughts, interfering with problem solving and positive instrumental behavior, and reducing sensitivity to changing circumstances (Nolen-Hoeksema et al., 2008; Offredi et al., 2016; Watkins and Roberts, 2020). In other words, such self-focused rumination often leads to maladaptive responses and chronic pain, resulting in impaired problem-solving skills and increased negative emotions, and may form a vicious cycle. It is crucial to have an in-depth comprehension of the neural mechanisms underlying rumination, which can contribute to enhancing our understanding of the etiology and mechanisms of these disorders, thereby increasing diagnostic accuracy and providing valuable insights for treatment and intervention.

An important feature of rumination is an intense self-focus (Watkins and Moulds, 2005), and this constant, self-referential cognitive activity is closely linked to the default mode network (DMN) (Berman et al., 2011; Hamilton et al., 2015; Rosenbaum et al., 2017; Zhou et al., 2020), which supports internal reflection and autobiographical memory (Buckner et al., 2008). The DMN can be further divided into three functionally distinct subsystems; researchers found that the subsystems of the DMN were not equally active during rumination and exhibited altered functional connectivity (Chen et al., 2020; Zhou et al., 2020). Another important feature of rumination is that it is associated with continuous negative emotions (Thomsen et al., 2003), which is related to brain regions involved in emotion regulation. For example, several studies have shown a significant correlation between rumination and activation of the amygdala (Ray et al., 2005; Mandell et al., 2014; Belzung et al., 2015). Furthermore, researchers found that depressed patients with high levels of rumination had reduced nodal centrality of the amygdala during task performance (Zhang et al., 2020). This suggests that the amygdala is unable to play a full role in emotion regulation in rumination, resulting in lack of regulation of emotional states. In addition, individuals tend to ignore changes in the external environment during rumination. As previous studies have found, the dorsal attentional network (DAN), which helps to focus and maintain attention to external stimuli, is negatively associated with rumination (Lois and Wessa, 2016; Rosenbaum et al., 2018a,b). Reduced functional connectivity has also been found between the frontoparietal network and DAN in depressed patients with high levels of rumination (Kaiser et al., 2015). To summarize, rumination is closely related to brain networks responsible for functions such as self-referential processing, attentional control, and emotion regulation.

While numerous studies have examined the effects of rumination, much less attention has been given to the factors that influence rumination. Rumination, as a complex cognitive process, can be influenced by a variety of factors, such as personality traits, genes, environmental conditions, events, and feedback modalities (Cox et al., 2010; Johnson et al., 2014; Watkins and Roberts, 2020). Although the social environment plays an important role in individual growth, few studies have investigated the effects of social relationships on rumination. Indeed, studies have shown that the negative effects of rejection are not limited to the emotional and psychological levels but may also extend to cognition and thought patterns (Voncken et al., 2008; Gunther Moor et al., 2010; Slavich et al., 2010; Rudert et al., 2021). For example, social rejection has been shown to lead to negative thought patterns (Monroe et al., 2007; Cacioppo and Hawkley, 2009). There is growing evidence that social rejection may influence individuals’ emotional and cognitive responses by eliciting ruminative thought processes, which may contribute to the development and maintenance of mood disorders (Goodman and Southam-Gerow, 2010; Wesselmann et al., 2013). In Williams (2009) temporal need-threat model, rumination is one of the events experienced after the occurrence of rejection. Additionally, researchers have proposed a psychobiological model of social rejection and depression, in which rejection-related events activate brain regions involved in processing negative emotions and pain, triggering negative self-referential thoughts and feelings of self-consciousness (Slavich et al., 2010), which are key features of rumination. Therefore, exploring the role of social rejection on rumination not only contributes to a deeper understanding of the effects of rejection on individuals but also provides new ideas for coping with its negative effects.

Recent advances in neuroimaging techniques have enabled researchers to investigate how brain functional connectivity is related to specific cognitive and affective processes. The connectome-based predictive model (CPM) is one such technique that has shown promise for predicting individual differences in cognitive and emotional traits (Shen et al., 2017). In this study, we used the CPM approach to identify a resting-state functional connectivity network that predicted rumination; we believed that this network would involve brain areas related to self-referential thoughts, emotions, and attention. Furthermore, we use mediation analyses to examine the role of this network in the relationship between social rejection and rumination. Our findings would provide novel insights into the neural mechanisms underlying the link between social rejection and rumination, and might have important implications for the development of interventions to mitigate the negative consequences of social rejection.

In summary, the present study aimed to demonstrate the followings: (1) social rejection is positively correlated with rumination, (2) brain functional connectivity features of rumination, and (3) social rejection-induced enhancement of ruminative traits (leading individuals to have a greater risk of anxiety and depression) through brain functional connectivity features.

## 2. Methods

### 2.1. Participants

The present study was conducted in a population of healthy adults, and none of them had a history of neurological or psychiatric illness (self-reported, with no history of brain damage, schizophrenia, major depression, anxiety disorder, and insomnia). All subjects in this study were from Southwest University, Chongqing, China. Psychological questionnaire and brain data were obtained from our ongoing project, Gene-Brain-Behavior (GBB). The GBB project aims to establish a multimodal database focusing on cognition and brain development to explore the relationships of structural and functional brain development with creativity, emotion, personality, etc.; these data have been used in several previous studies (Chen et al., 2019; Zhuang et al., 2021). The research protocol was approved by the Ethics Committee of the Brain Imaging Center of Southwest University. All participants signed an informed consent form prior to the data acquisition and were paid 50 RMB for their participation. Of the subjects recruited for this study, 593 had complete data available for psychological measures that were able to be matched with brain data. Thirteen subjects were excluded due to excessive head movement (mean head movements >0.3 mm), and 20 subjects were excluded due to substandard brain imaging data (artefacts, etc.). Thus, data from 560 subjects (mean age: 19.32 ± 1.27 years; range: 16–26 years; 401 females and 159 males) were included in the analyses. Basic details of the participants, along with additional information, are available in the Supplementary material.

### 2.2. Psychological questionnaire

Rumination was measured with the Ruminative Responses Scale (RRS) (Nolen-Hoeksema and Morrow, 1991). The RRS contains 22 items rated a scale from 1 (almost never) to 4 (almost always) regarding the frequency of ruminative thoughts (e.g., Think “Why do I have problems other people do not have?”). The RRS comprises three subscales: reflective pondering, brooding and depression-related. We removed the third subscale, depression-related responses, as in previous studies (Li et al., 2022), because we focused on the behavioral of ruminative thoughts rather than its emotional consequences and because of the spurious discriminatory validity given its overlap with symptoms of depression. Thus, the final rumination score encompassed two subscales (reflective pondering and brooding) and had a Cronbach’s alpha coefficient of 0.92 (Lackner and Fresco, 2016).

Social rejection was assessed using the Perceived Rejection survey from the NIH Toolbox on Emotion (Salsman et al., 2013). On this survey, participants rate the 8 items on a scale from 1 (never) to 5 (always) regarding their feelings of rejection (e.g., “Does not listen when I ask for help”). The Cronbach’s alpha coefficient for the Perceived Rejection survey was 0.93 (Salsman et al., 2013).

### 2.3. Image data acquisition and preprocessing

The resting-state scan lasted 8 min, during which participants were told to keep their bodies as still as possible, not to think about specific things, and to keep their eyes open as much as possible. During the scan, a lab assistant was present outside the scanning room to monitor the participant’s state and respond to his or her needs.

Functional and structural data were obtained using a Siemens 3 T Trio scanner (Siemens Medical System, Erlangen, Germany) at the Brain Imaging Center of Southwest University. Resting-state fMRI data were obtained using a gradient-echo echo-planar imaging (GRE-EPI) sequence with the following parameters: repetition time (TR) = 2,000 ms, echo time (TE) = 30 ms, flip angle (FA) = 90°, field of view (FOV) = 220 × 220 mm^2^, slices = 32, thickness = 3 mm, interslice gap = 1 mm, and voxel size = 3.4 × 3.4 × 4 mm^3^. A high-resolution, three-dimensional magnetization-prepared rapid acquisition gradient echo (MPRAGE) sequence with the following parameters was conducted: TR = 1,900 ms, TE = 2.52 ms, FA = 9°, slices = 176, FOV = 256 × 256 mm^2^, thickness = 1 mm, and voxel size = 1 × 1 × 1 mm^3^. Images were preprocessed using the CONN toolbox (Whitfield-Gabrieli and Nieto-Castanon, 2012). Slice timing correction was applied. During head motion estimation and correction, we also regressed out outlier scans due to head motion. Potential outlier scans were identified based on two criteria: the observed global BOLD signal and the amount of subject motion during scanning. Acquisitions with framewise displacement above 0.9 mm or global BOLD signal changes above 5 s.d. were flagged as potential outliers. The images were segmented into grey matter, white matter (WM), and cerebrospinal fluid (CSF) and normalized to standard MNI space (Ashburner and Friston, 2005). An 8-mm full-width at half-maximum Gaussian kernel was applied to smooth the images. The images were then denoised using the anatomical component-based correction (aCompCor) method, and we regressed out signals from WM, CSF, and 12 head movement parameters. After denoising, the images were linearly detrended and bandpass filtered (0.01–0.1 Hz). Global signal regression was not used in this study, as hemispheric segregation and hemispheric integration are based on the functional connectivity (FC) between these regions and corresponding homotopic regions.

### 2.4. Functional network construction

FC matrices were constructed using the GRETNA toolbox (Wang et al., 2015a). The atlas used to calculate FC in this study partitioned the cortex into 400 ROIs and grouped them into 7 major brain networks with specific functions: the visual network (VIS), somatomotor network (SMN), dorsal attention network (DAN), salience network (SAL), limbic network (LIM), frontoparietal control network (FPCN) and default mode network (DMN)(Schaefer et al., 2018). In constructing the functional connectivity matrix, the time series data of the node were extracted from each ROI, and the “edges” were represented by the Pearson correlation coefficients of the time series of the nodes, which underwent a Fisher z transformation (Zar, 1999). The significance of the negative values of the resting-state functional connectivity matrix is not clear and is susceptible to preprocessing methods and noise, which may be artificially created false correlations that do not necessarily indicate negative coupling between regions (Murphy et al., 2009; Chai et al., 2012).Therefore, negative values in the matrix were set to zero as previous studies (Chan et al., 2014, 2021; Wang et al., 2021a, 2022). Each subject’s data were constructed as a 400 × 400 matrix for further analysis.

### 2.5. Connectome-based predictive modeling

**Figure 1:**
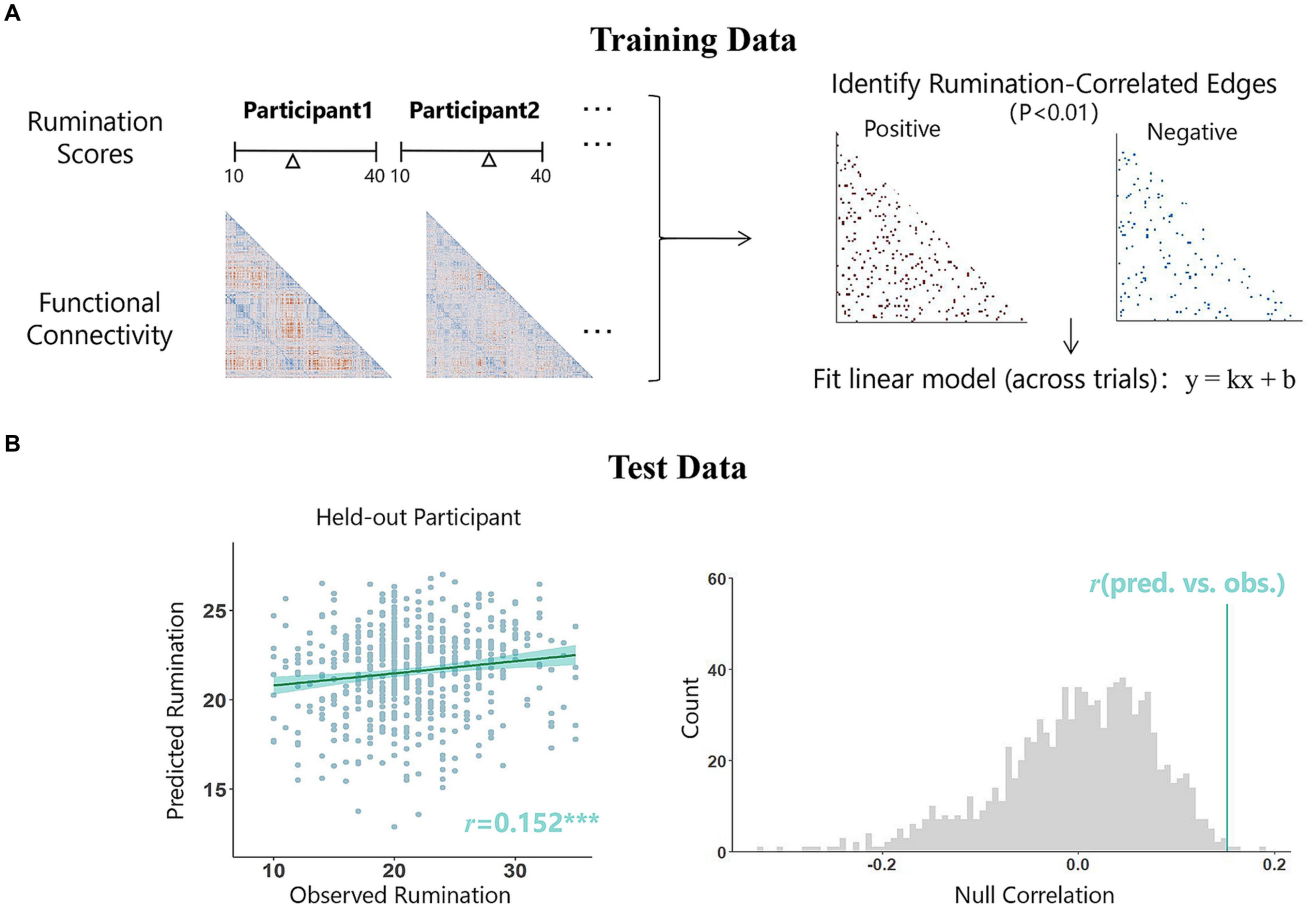
Connectivity-based predictive model of rumination. **(A)** For the leave-one-out method, in each iteration, data from *N*-1 participants are used as the training data, while the data from the remaining individual serve as the testing data. Within the training data, we calculated the correlation coefficients between resting-state functional connectivity and rumination scores and selected the significant edges (*p* < 0.01), the values of all these edges in each network were summed to obtain the strength of the prediction network. Next, the general linear model was trained to reflect the relationship between prediction network strength and rumination scores. This model will then be used in the test set to obtain a predicted score. This process will be repeated N times until each participant has received a prediction score. Edges retained each iteration form the final prediction network (red: positive correlation; blue, negative correlation). **(B)** The predictive efficacy of the model was judged by calculating the Pearson’s correlation coefficient between the predicted scores and the observed scores. This correlation value (indicated by the blue line) was then compared with the null distribution of *r* values from 1,000 random permutations. The scatterplot on the left side of the figure demonstrates the relationship between the predicted scores obtained using the negative prediction network and the actual scores in this study, while the right side presents the results of the permutation test, *p_perm_* = 0.004.

Here, we used a leave-one-out approach of connectome-based predictive modeling (CPM) (Shen et al., 2017) to predict rumination from rs-fMRI connectivity, which has been widely used in recent years to predict anxiety (Wang et al., 2021b), irritability (Scheinost et al., 2021), loneliness (Feng et al., 2019), etc. CPM consists of three steps: feature selection, model construction, and model validation (Ren et al., 2021). First, we calculated the correlation coefficients between resting-state functional connectivity and rumination scores and selected the significant edges (*p* < 0.01)(Kucyi et al., 2021; Scheinost et al., 2021), including both positive and negative edges. The retained edges were used to form the positive and negative prediction networks. For leave-one-out cross-validation, one participant was excluded from the training set in each iteration, and the model was trained with data from N-1 participants (i.e., 559 in this study). Then, data from the excluded participant were used as a validation set to obtain the predicted value, and this process was repeated until each participant had obtained one. Thus, a positive network and a negative network were obtained in each iteration, and the final prediction network consisted of edges that appeared in each iteration (Figure 1A). Next, the values of all these edges in each network were summed to obtain the strength of the prediction network. The general linear model (GLM) was trained with these data to reflect the relationship between prediction network strength and rumination. The predictive efficacy was reflected by the Pearson correlation between the predicted and observed scores. If a relatively strong correlation was observed, the prediction model was successful. We further controlled for covariates such as age, sex, and mean head movement to determine whether these factors had a confounding effect (see at Supplementary material). Finally, we performed an iterative test (1,000 iterations) to obtain the distribution of the observed correlation coefficients (Figure 1B). The number of times that the distribution was greater than the true value was divided by the number of permutations to calculate the *p* value.

### 2.6. Model validation

To enhance the rigor of our study and attain more robust outcomes, we employed the ten-fold cross-validation method for model validation. In contrast to leave-one-out method, the adoption of ten-fold cross-validation involves a more stringent evaluation, mitigating the randomness introduced by specific data partitioning and better controlling the risk of overfitting. In this method, we randomly divided all 560 participants into 10 groups. The model was trained using data from 9 of these groups and tested with data from the remaining group; this process was repeated for all groups. Here, we used the same significance threshold as the leave-one-out approach above (*p* < 0.01). Since random grouping may introduce bias regarding model prediction efficacy, we repeated this process 100 times to reduce the effect, after which we obtained the averaged r and *p* values. Then, we performed 1,000 random permutations to generate the null distribution for significance testing.

### 2.7. Mediation analysis

We conducted a mediation analysis to explore whether rumination network strength mediated the association between social rejection and rumination scores. Analyses were conducted using Model 4 of the indirect macro PROCESS designed for SPSS (Preacher and Hayes, 2008). In the present study, the independent variable (*X*) was the social rejection score, the dependent variable (*Y*) was the rumination score, and the mediation variable (*M*) was the strength of the prediction network. Indirect effects were considered significant if zero was not included in the bootstrapped 95% confidence intervals (CIs) (5,000 samples).

## 3. Results

### 3.1. Psychological questionnaire results

We verified the completeness, logical consistency, and reasonable timing of the collected questionnaires, assessed the rationality of data distribution, and examined the presence of outliers and non-genuine responses. At this stage, no participants were excluded. We used Pearson’s correlation coefficients to explore the correlation between social rejection and rumination, and the results showed that there was a significant positive correlation (*r* = 0.283, *p* < 0.001).

### 3.2. CPM Results

#### 3.2.1. Predictive efficacy of the leave-one-out approach

**Figure 2:**
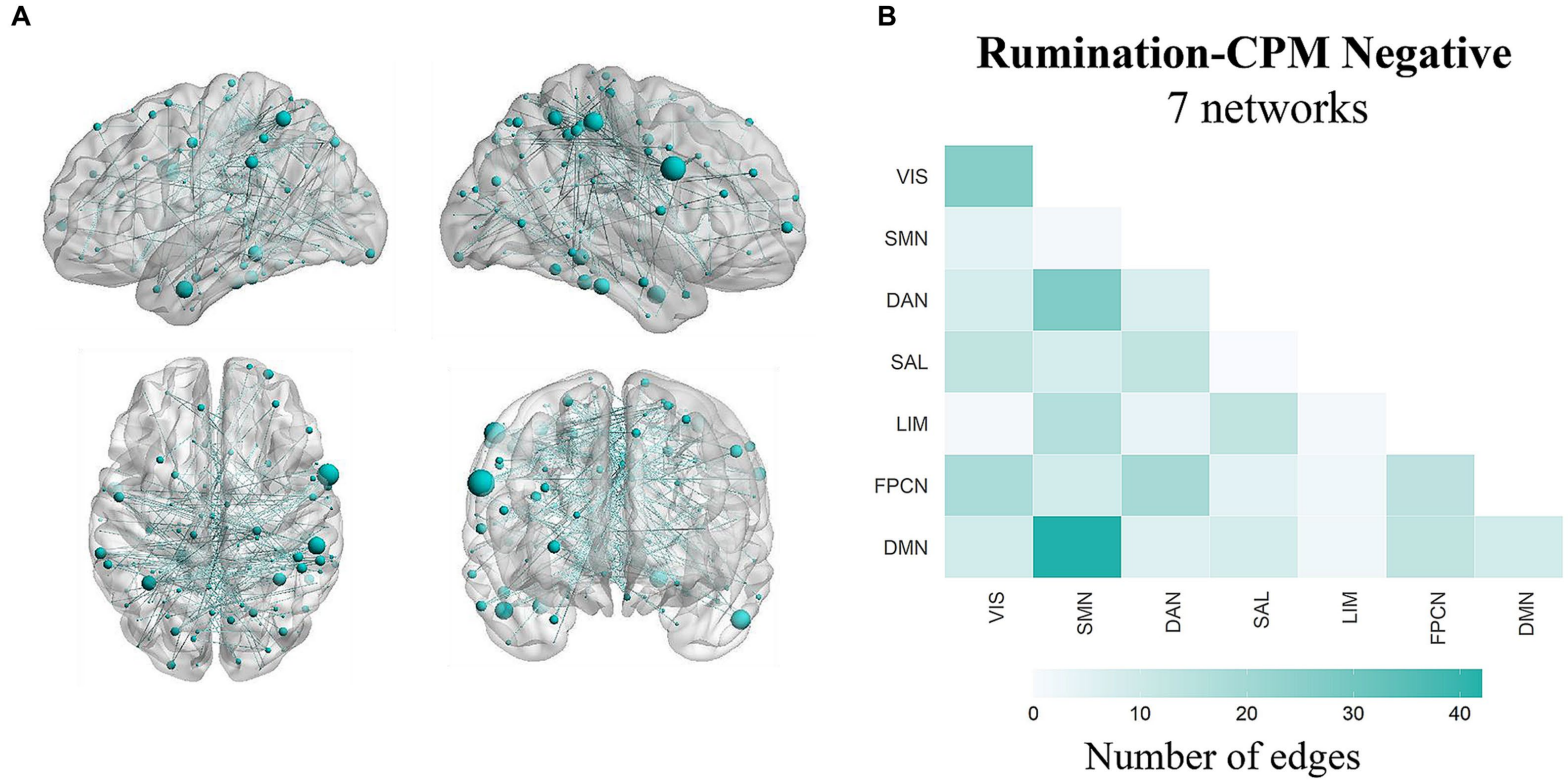
Functional neuroanatomical basis of the predictive network. **(A)** Edges from the negative prediction network. Only edges that appeared in each iteration were retained. **(B)** The number of edges in the negative prediction network assigned to each intra- or internetwork pair based on the Schaefer400 and Yeo-Krienen 7-network atlases. VIS, visual network; SMN, somatomotor network; DAN, dorsal attention network; SAL, salience network; LIM, limbic network; FPCN, frontoparietal control network; DMN, default mode network.

The network strength of a positive or negative prediction network is obtained by summing the values of all edges within the network. Predictive efficacy was represented by the correlation between the observed rumination scores and predicted rumination scores. After leave-one-out cross-validation, there were 182 edges in the positive network and 299 edges in the negative network (Figure 2A). The results showed that the negative network significantly predicted individual differences in rumination scores (*r* = 0.152, *p* < 0.001, *p_perm_* = 0.004), while the positive network did not significantly predict these differences (*r* = 0.045, *p* = 0.288). The *p_perm_* values was based on permutation testing (1,000 permutations). Controlling for head movement, sex, and age did not affect the validation of the results.

#### 3.2.2. Functional neuroanatomical basis of the predictive network

To better understand the functional neuroanatomical implications of the prediction network, we assigned the 299-edge negative network into intranetwork and internetwork connections according to the Schaefer400 atlas (Schaefer et al., 2018). The Schaefer400 atlas can classify nodes into 7 networks or 17 networks; here, we used the 7-network classification, consistent with the Yeo-Krienen networks (Yeo et al., 2011). Similar to the findings of previous studies (Chen et al., 2020), the DMN was closely associated with rumination and contributed strongly to the prediction of rumination among the networks. The top five network pairs contributing to negative edges were as follows: DMN-SMN, DAN-SMN, VIS–VIS, FPCN-DAN, and FPCN-VIS (Figure 2B).

**Figure 3:**
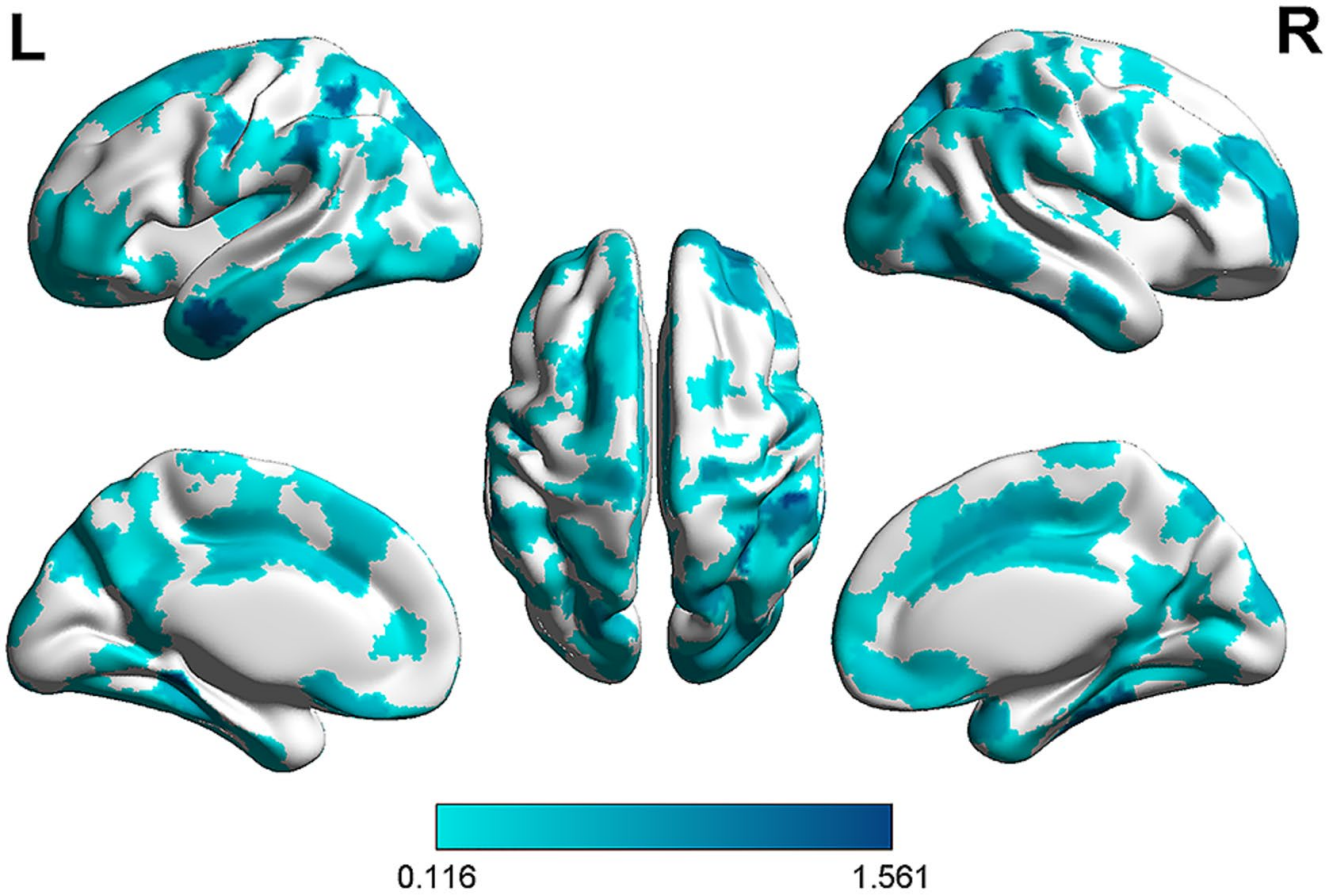
The strength of nodes in the prediction network. Edge weights were obtained by calculating the correlation coefficients between rumination scores and functional connectivity in the prediction network. Node strength was computed by summing the absolute value of the correlation coefficients. The darker the color, the greater the weight of the ROI in the predictive model, indicating a stronger association with rumination.

To further understand the importance of each node within the prediction network, we obtained the node strength by calculating the sum of the edge weights connected to the node (He et al., 2021). Specifically, weights were correlation coefficients between the functional connectivity and rumination scores. Then, we summed the absolute values of these correlation coefficients (since all correlation coefficients were negative) to obtain the strength of these nodes. The results showed that regions with higher node strength were mainly located in the supramarginal gyrus, angular gyrus, middle temporal gyrus, 3 inferior temporal gyrus, fusiform gyrus and parahippocampal gyrus (Figure 3).

#### 3.2.3. Model validation

Tenfold cross-validation yielded similar results as the leave-one-out method. The negative network significantly predicted rumination scores (*r* = 0.125, *p* = 0.005, *p_prem_* = 0.016), while the positive network did not.

### 3.3. Mediation analysis

**Figure 4:**
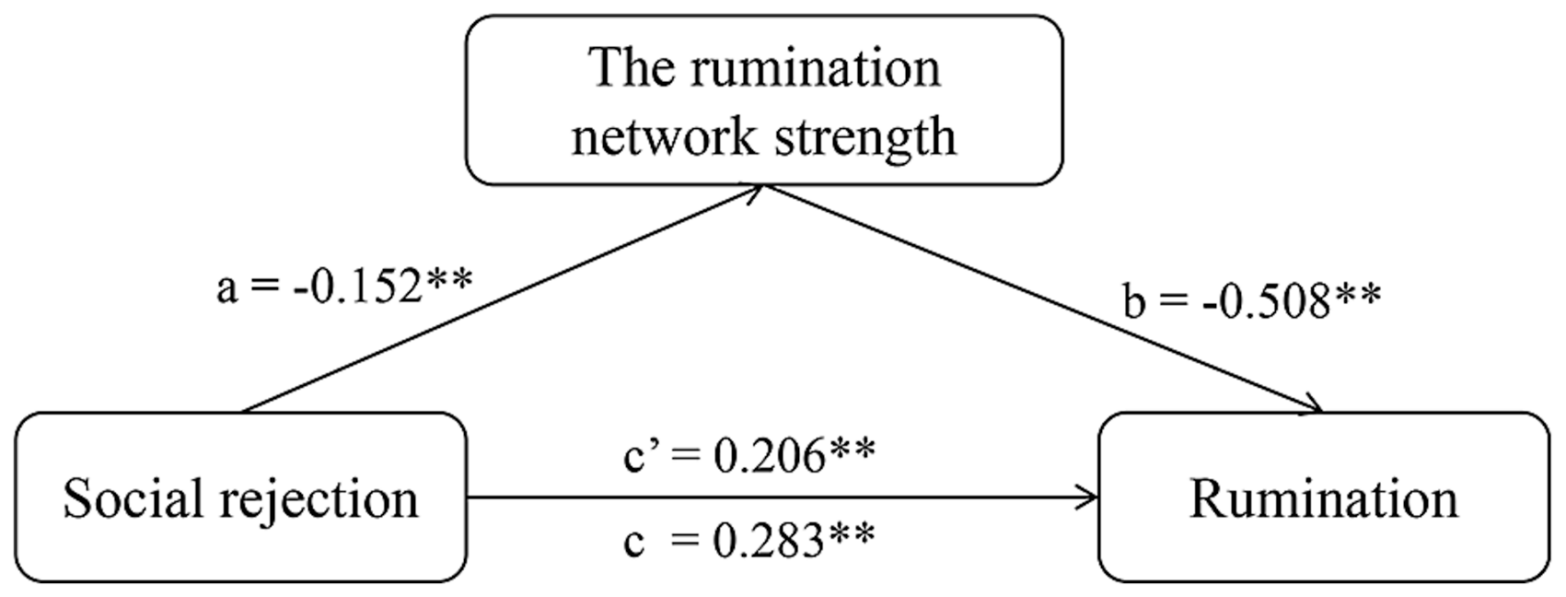
The mediation model of the relationships among social rejection, rumination network strength, and rumination. Mediation analysis is a method used to explain the mechanism through which an independent variable influences a dependent variable via an intermediate variable. In the present study, the independent variable (*X*) was the social rejection scores, the dependent variable (*Y*) was the rumination scores, and the mediation variable (*M*) was the strength of the prediction network. Path a represents the influence of *X* on *M*. Path *b* represents the influence of the *M* on the *Y*. Path *c*, the total effect, includes both the direct and indirect effects, representing the overall impact of *X* on *Y*. Path *c’* represents the direct impact of *X* on *Y* when not considering the mediator variable *M*. *p < 0.05; **p < 0.01.

We defined the sum of the FC values as the strength of the prediction network. In the mediation analysis, the independent, mediating, and dependent variables were social rejection, prediction network strength, and rumination. The relationship between social rejection and rumination (*c’* = 0.206, *p* < 0.001) was mediated by prediction network strength (*a* = −0.520, *p* < 0.001; *b* = −0.508, *p* < 0.001). The 95% confidence interval of the mediation analysis did not include 0 (LLCI = 0.038, ULCI = 0.121), indicating that this indirect effect was significant (Figure 4).

## 4. Discussion

Rumination has received widespread attention as an important factor in the maintenance of a variety of psychiatric disorders (Watkins and Roberts, 2020). However, to date, no studies have constructed predictive models of rumination from the functional connectivity characteristics of healthy individuals. In addition, research has consistently focused on the effects of rumination rather than the factors that influence rumination. In the present study, we established a resting-state functional connectivity network capable of predicting rumination scores using CPM and assessed the validity of the prediction model using two different cross-validation methods (leave-one-out and tenfold cross-validation). The model maintained predictive validity after controlling for sex, age and mean head movement. We then calculated the strength of the prediction network by summing the values of all FC edges and verified its mediating role in the relationship between social rejection and rumination. These results suggest that social rejection may lead to more rumination by affecting functional networks in the brain.

The present study assigned functional connectivity values to a functionally explicit large-scale network to further investigate the neural basis of the prediction network; the results indicated that the following networks strongly contributed to the predictive power of the network: the default mode network (DMN), dorsal attention network (DAN) and frontoparietal control network (FPCN). The DMN is one of the networks that has received the most attention regarding the cognitive mechanisms of rumination due to its high relevance to self-awareness (Davey et al., 2016). Studies have demonstrated that the DMN is involved in self-referential processes, theory of mind and autobiographical memory (Buckner and Carroll, 2007; Sheline et al., 2009; Qin and Northoff, 2011). A task-state fMRI study found that the overall FC within the entire DMN tended to decrease during rumination, including FC between the core and dorsal medial prefrontal cortex (dmPFC) subsystems (Chen et al., 2020). The dmPFC subsystem is widely believed to be associated with mentalizing and present-oriented thoughts (Van Overwalle, 2011; Wagner et al., 2012; Andrews-Hanna et al., 2014). Therefore, our results may indicate that during rumination, people tend to devote more attention to past events and thus less attention to their present state. Notably, rumination is a multifaceted process and its link to DMN cannot be simply defined. Since individuals may engage differently with their past experiences and present states during rumination, it is essential to recognize that changes in FC patterns can be context-dependent and influenced by various factors (Zhou et al., 2020; Tsuchiyagaito et al., 2021). Future research may be able to explore the relationship between the different cognitive processes involved in rumination state and DMN connectivity by adopting more fine-grained approaches.

Extensive experiments have shown that the DAN supports the maintenance of concentration, allowing people to focus on the same thing for longer periods of time and ignore external influences (Corbetta and Shulman, 2002; Corbetta et al., 2008), while the FPCN supports executive control of attention and emotions, playing an important role in inhibitory control and cognitive flexibility (Bressler and Menon, 2010; Zhang et al., 2017; Kupis et al., 2021). Dixon et al. (2018) found that one subsystem in the FPCN regulates mental activities such as introspection through connections with the DMN, while the other subsystem is connected to the DAN and regulates processes such as top-down attentional selection. Therefore, decreased FC within and between these networks predicts less attention to the external environment and stimuli, during which time individuals’ cognitive control may be compromised, leading to a greater likelihood of dwelling on recurring memories and suffering negative emotions.

In addition, the SMN also contributed strongly to the prediction model. A number of previous studies have identified that the SMN plays a role in the perception of external stimuli, body awareness and emotional and cognitive processing (Pineda, 2008; Banissy et al., 2010; Wood et al., 2016; Davis et al., 2017). In addition, decreased FC between the SMN and the DAN might underlie attention deficits (Wang et al., 2015b; Zhu et al., 2016). Studies on bipolar disorder have suggested that changes in connectivity between the SMN and DMN may be related to impaired emotion processing and executive function (Martino et al., 2016; Kazemi et al., 2018; Zhu et al., 2022). This may indicate that impaired cognitive control during rumination makes it difficult for individuals to shift their attention away from negative stimuli and that this continuous processing of self-relevant negative events leads to persistence of ruminative thoughts.

Analysis of node strengths revealed that key nodes predicting rumination scores were located in the supramarginal gyrus (SMG), angular gyrus (AG), middle temporal gyrus (MTG), inferior temporal gyrus (ITG), fusiform gyrus (FFG) and parahippocampal gyrus (PHG), areas which are closely related to memory, attention and emotion. The SMG supports attention orientation, emotion regulation, language and verbal working memory (Rushworth et al., 2001; Silk et al., 2010; Deschamps et al., 2014), and researchers have found that the SMG is also capable of integrating perceptual information from multiple modalities and is associated with self-related emotions (Silani et al., 2013). The AG supports memory retrieval, attention and spatial cognition, semantic memory, reasoning, and other higher-order cognitive functions (Seghier, 2013; Humphreys et al., 2021). The MTG is activated by processes regarding memory and emotion (Chan et al., 2009; Fan et al., 2017), and some studies have suggested that it may be related to poor emotion regulation and neuroticism (DeYoung et al., 2010; Zhang et al., 2022). In addition, the ITG and the FFG are brain regions closely associated with the processing of perceptual information (Rossion et al., 2003; Domínguez-Borràs et al., 2009), such as visual and auditory information, and FFG has also been found to play a crucial role in face processing (Rossion et al., 2000).While the PHG is related to emotion regulation and autobiographical memory processes (Almeida et al., 2009; Frank et al., 2014).

The results from the questionnaire analysis showed that social rejection and rumination were positively correlated. Furthermore, we found that the prediction network played a mediating role in the relationship between social rejection and rumination. Rejection damages interpersonal relationships and threatens basic needs such as sense of belonging and security (Kemeny, 2009). Once individuals experience rejection, they suffer pain, negative emotions, and lack of satisfaction of their basic needs, leading them to focus on the experience of rejection and continually recall, consider and assess the meaning and importance of the rejection event (Williams, 2009). After suffering social rejection, people were more inclined to respond by withdrawing from social contact, hoping to protect themselves from further social loss. This withdrawal was accompanied by rumination, which may represent heightened motivation to prevent similar circumstances (Molden et al., 2009). However, studies have shown that rumination may also occur after interpersonal difficulties lead to reduced satisfaction with our relationships, leading individuals to become less confident, rejection sensitive, exhibit maladaptive responses and reduced interpersonal problem solving, ultimately preventing them from recovering from rejection (Lam et al., 2003; Kuehner and Buerger, 2005; Pearson et al., 2010; Wesselmann et al., 2013). Numerous studies have shown that the DMN is broadly related to social cognition and is coupled with various other networks, such as the attention network, during different social cognition tasks (Schurz et al., 2014; Xie et al., 2016; Schurz et al., 2020). In the present study, the SMG and the AG contributed the most to the prediction network. A study showed that the SMG is associated with emotional egocentric bias (Silani et al., 2013), and we hypothesized that social rejection may trigger self-referential rumination by triggering emotional experiences strongly related to the self. The AG is a region in the core system in the default mode network associated with functions from mentalization to semantic processing and memory retrieval; this region is responsible for detecting behaviourally relevant stimuli and environmental changes (Seghier, 2013; Spreng and Andrews-Hanna, 2015). The mediation analysis results may explain the tendency of people to immerse themselves in introspection after rejection, decreasing their cognitive load during other cognitive tasks and overlooking changes in the environment. In conclusion, we hypothesize that social rejection may predispose people to rumination by inducing more assessment of self- and social-related events as an emotion-regulation strategy for coping with the negative experience of rejection.

There are still some limitations of our study. First, we explored the influence of social relationships on rumination; therefore, we chose social rejection, which is one of the most important characteristics in social relationships; however, such data are inevitably subjective in nature. Subsequent experiments could attempt to use other approaches, such as the construction of social networks, to utilize objective data. Second, this study examined the relationship between social rejection and rumination from a cross-sectional perspective, without further exploring the wider consequences of rumination on social cognition and relationships. Follow-up studies should further explore the possible long-term relationship between rumination and rejection with longitudinal designs as well as the impact of this vicious circle on depression and anxiety. Besides, at the stage of constructing the functional connectivity matrix, we excluded negative values as their meaning was unclear, which could have resulted in the loss of valuable information. Finally, the results of this study inspired us to consider new approaches to intervening rumination from a social perspective. Future research could explore the effectiveness and feasibility of interventions, such as providing mental health education and social support, offering training in emotion regulation skills and social skills, and implementing socio-emotional interventions.

## 5. Conclusion

This study was the first to develop a network capable of predicting rumination scores that was trained with resting-state functional connectivity data from a healthy population. We found that the functional connectivity among the DMN, DAN, FPCN and SMN contributed strongly to the predictive model. We explored the relationship between social rejection and rumination, demonstrating that the experience of rejection may lead to rumination. These findings enhance understanding of the impact of social rejection and can inform the development of interventions for rumination from a social cognitive perspective.

## Data availability statement

The raw data supporting the conclusions of this article will be made available by the corresponding author. Requests to access the datasets should be directed to author JQ at qiuj318@swu.edu.cn.

## Ethics statement

The studies involving humans were approved by the Brain Imaging Center Institutional Review Board at the Southwest University, China. The studies were conducted in accordance with the local legislation and institutional requirements. The participants provided their written informed consent to participate in this study.

## Author contributions

LG: Conceptualization, Methodology, Validation, Visualization, Writing – original draft, Writing – review & editing. QF: Conceptualization, Methodology, Validation, Visualization, Writing – review & editing. XW: Methodology, Software, Validation, Visualization, Writing – review & editing. YG: Methodology, Software, Writing – review & editing. LH: Conceptualization, Software, Writing – review & editing. JQ: Conceptualization, Data curation, Funding acquisition, Supervision, Writing – review & editing.
