# Supplementary material for: Connectome-based modeling reveals a resting-state functional network that mediates the relationship between social rejection and rumination
Source: Front Psychol. 2023 Oct 30;14:1264221. doi: 10.3389/fpsyg.2023.1264221 (PMC10642796; doi:10.3389/fpsyg.2023.1264221)
Supplement: Supplementary file 1 [file Table_1.DOCX]

Supplementary Information

**Connectome-based modeling reveals a resting-state functional network that mediates the relationship between social rejection and rumination**

Geng et al.

**Supplementary Methods**

# **Gene-Brain-Behaviour (GBB) Project**

Gene-Brain-Behaviour (GBB) project aims to establish a multimodal database focusing on cognition and brain development to explore the relationships of structural and functional brain development with creativity, emotion, personality, etc. GBB extensively recruited healthy, right-handed adult college students, and have completed multimodal data collections from nearly 1,000 individuals. Participants completed a wide range of psychological measures during the data collection process, including assessments of intelligence, personality, creativity, mood, and social factors. Additionally, scans of brain structure and functional imaging were performed. It is worth noting that all participants underwent assessments for brief psychiatric disorders, which included depression scores measured using several different questionnaires, such as the Symptom checklist-90(SCL-90), Beck Depression Inventory-I(BDI-I), Beck Depression Inventory-II (BDI-II), and Center for Epidemiological Survey-Depression Scale(CES-D).

The GBB project followed these steps in the data collection process: Firstly, using online postings and posters to recruit healthy adult college students. Next, obtain their demographic information after which participant could get an individual subject number. Subsequently, participants underwent brain imaging data acquisition, encompassing both structural and functional brain images. All participants underwent T1 and resting-state scans. Furthermore, some participants also performed cognitive tasks during the scanning (not related to the present study). After brain data acquisition, participants completed various psychological assessments related to cognition, affect, and personality during multiple visits. Notably, the two questionnaires used in this study were administered during the same visit.

# **Participants**

The details of the 20 participants who were excluded due to substandard brain imaging data are as follows:

Seven participants were deemed ineligible for data inclusion because they failed to complete the scanning process correctly. Among these, three participants fell asleep during scanning, one reported excessive noise that prevented him from remaining calm and following instructions, and three participants made repeated noises and asked questions during the scan.

Additionally, nine participants were excluded due to excessive artifacts, two participants had incomplete data saved after the scanning process due to equipment issues, and one subject was excluded due to abnormal brain structure.

**Supplementary Results**

**Participant Psychological Characteristics Statistics**

**Supplementary Table 1: Scores for Participants on Rumination, Social Rejection, and Depression.**

| Measures | Mean | Standard Deviation | Median | Range |
| --- | --- | --- | --- | --- |
| Rumination Score (Total Score of Three Subscales) | 44.63 | 10.89 | 44 | 22-83 |
| Reflection pondering (R) | 10.48 | 2.89 | 10 | 5-20 |
| Brooding (B) | 11.09 | 2.91 | 11 | 5-20 |
| Depression-related | 23.06 | 6.15 | 23 | 12-45 |
| Non-Depression-Related Rumination Score (used in present study) | 21.57 | 5.36 | 21 | 10-39 |
| Social Rejection | 16.14 | 5.16 | 16 | 8-38 |
| Beck Anxiety Inventory | 27.06 | 5.73 | 25 | 21-63 |
| Beck Depression Inventory-II | 6.98 | 6.32 | 5 | 0-40 |
| Center for Epidemiological Survey-Depression Scale | 14.77 | 7.90 | 14 | 0-50 |

**CPM Analysis with Negative-Valued Functional Connectivity**

Due to the ambiguous interpretation of negative values in resting-state functional connectivity, we defined negative values in the functional connectivity matrices as zero in the primary analysis. However, such an operation might lead to the loss of valuable information. Therefore, we conducted a control analysis that included all functional connectivity data.

The results revealed that the negative prediction model remained statistically significant (*r* = 0.112, *p* = 0.008). The inclusion of all FC edges increased the number of features from the original 299 edges to 318 edges. Unfortunately, the addition of these features did not lead to an improvement in the predictive performance of the model. Instead, it resulted in a decrease in predictive accuracy when compared to the original model (*r* = 0.152). In addition, the positive prediction model remains non-significant (*r* = 0.041, *p* = 0.328).

**Control analysis of CPM**

After accounting for potential confounding variables, including gender, age, and mean head movement, our predictive model continued to demonstrate its validity. Consistent with the primary findings of this study, the negative prediction network remained significantly correlated with rumination (*r* = 0.176, *p* < 0.001) even after controlling for these additional factors. Conversely, the positive prediction network remained statistically insignificant (*r* = -0.028, *p* = 0.512). A visual representation of the predicted scores versus actual scores can be found in Supplementary Figure 1.

**
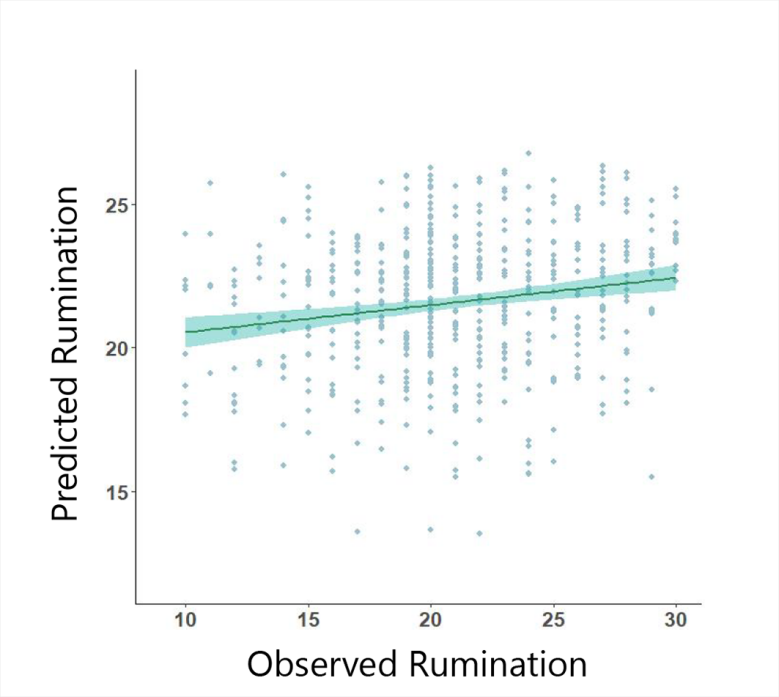
**

**Supplementary Figure 1**  Correlations between predicted rumination scores and actual rumination scores after controlling for gender, age, and mean head movement.
